# Factors determining nutrient distribution in sediments and porewater in a semi-confined coastal environment subject to anthropogenic pressure

**DOI:** 10.1007/s10661-026-15065-y

**Published:** 2026-02-23

**Authors:** Gabriela Cugler de Pontes, Susanne Schmidt, Murilo de Carvalho Vicente, Teresa Cristina Guimarães, Wilson Thadeu V. Machado, Julio Cesar Wasserman

**Affiliations:** 1 Post-Graduate Program in Geochemistry, University Federal Fluminense, Niterói, Brazil; 2 Master’s Program Environmental Science and Technology, Technical University Berlin, Berlin, Germany; 3 Department of Geoenvironmental Analysis, University Federal Fluminense, Niterói, Brazil

**Keywords:** Nutrient storage, Interstitial water, Nitrogen, Phosphorus, Guanabara Bay–Brazil

## Abstract

Increasing inputs of nutrients and organic matter from urban areas accumulate in coastal sediments, which act as a storage compartment that can trigger eutrophication events, and promote organism mortality. The aim of this research was to evaluate the factors that determine the distribution of nutrients in sediments and porewater from a coastal environment. Jurujuba cove receives nutrients and organic matter inputs from diffuse sources (mainly domestic sewage) and from intensive mussel farming. Fifteen sediment samples were collected. pH and Eh were measured in situ. Sediments were centrifuged to extract porewater, where dissolved nutrients were analyzed. Total phosphorus and total organic carbon were analyzed in the bulk sediment by spectrophotometry and titration, respectively. Granulometry was analyzed with a laser granulometer, and carbonates were measured gravimetrically. Sediment phosphorus concentrations ranged between 0.2 and 3.2 mg g^−1^ and were possibly related to sewage and aquaculture, with higher concentrations at the Cachoeira River mouth and close to mussel farms. The results indicated high dissolved nitrogen (2.2 ± 4.8 mg L^−1^) and phosphorus (0.3 ± 0.3 mg L^−1^) concentrations in porewater, indicating possible diffusive release to the water column. The Q-mode cluster analysis showed the formation of two distinct groups, determined by the characteristics of the sediments. The first group incorporates samples located near the shoreline, representing an environment with higher accumulation of organic matter (average 16.6%). The other group comprised stations in the middle of the inlet, showing slightly smaller concentrations of organic matter (average 12.0%). Nonetheless, overlapping physicochemical conditions made it difficult to clearly separate the influence of urban drainage and mussel farming, indicating that multiple stressors act simultaneously in the cove. The application of isotopic relationships (nitrogen mainly) would contribute to the depiction of sources in the research area.

**Supplementary Information:**

The online version contains supplementary material available at 10.1007/s10661-026-15065-y.

## Highlights

- Nutrient behavior in the porewater controls water quality.
- Eh determines nutrient and organic matter retention/release from sediments.
- Breaking waves modify physicochemical conditions and affect nutrients on the shoreline.
- Nutrient inputs from drainages are relevant, but do not always determine enrichment.
- Mussel farms reduce hydrodynamics, leading to nutrient accumulation.

**Supplementary Information:**

The online version contains supplementary material available at 10.1007/s10661-026-15065-y.

## Introduction

Coastal sediments record pollutant inputs (Tarique et al., 2013) and associated physicochemical shifts. In reducing depositional environments, the dissolution of ions enriches the porewater, creating a reservoir of dissolved species (Quevauviller, 2016). These species can subsequently flow to the overlying water column via molecular diffusion, advection, and sediment resuspension (Baumgarten & Niencheski, 2010; Kim et al., 2016; Zhang et al., 2004). Thus, biogeochemical processes within the sediments directly affect water quality by regulating the release of legacy or newly mobilized contaminants (Friedrich et al., 2003; Nowlin et al., 2005).

Biogeochemical processes can alter the physicochemical properties of the sediments. An example is pH, which is regulated by the dissociation of dissolved CO_2_ into bicarbonate -HCO_3_^−^ (Todd Schaef & Peter McGrail, 2005), and strongly influenced by primary production, removing CO_2_ from the water column (Lendt et al., 2003). Shifts in pH and redox conditions, often coupled with carbonate dissolution, can subsequently increase cation mobility (Brady & Weil, 2008). Simultaneous microbial activity drives the anaerobic degradation of organic matter, primarily through seawater sulfate reduction and remineralization of nutrients in the porewater (Gieskes et al., 2015). The dynamics of these interconnected processes are critical, as they ultimately control the trophic state of aquatic systems (Vicente et al., 2021).

In low-energy depositional environments, which are characterized by fine-grained sediments and reduced hydrodynamics, anaerobic conditions readily develop. These stable conditions favor the preservation and accumulation of organic matter (Zeng et al., 2011). Under these conditions, low oxidation and less intense anaerobic fermentation promote conservation of organic matter, which is enriched in the sediments. The result is a suitable environment for the development of nutrient remineralization, leading to eutrophic conditions (Nixon, 1995). Furthermore, the slow currents of such environments reduce the water turnover time (Knoppers et al., 1991), amplifying the effects of nutrient enrichment, sometimes developing hypertrophic conditions.

Anthropogenic nutrient inputs exacerbate this cycle by stimulating high rates of primary production. The subsequent decay of this organic matter generates a strong biological oxygen demand (BOD), leading to hypoxia or anoxia. These processes result in the degradation of the biological diversity, creating “dead zones” (McQuatters-Gollop et al., 2009). Remediation is particularly challenging in such settings, as nutrient-rich sediments become a persistent internal source, perpetuating eutrophication (Fathollahzadeh et al., 2015; Scholes & McIntosh, 2010).

In many choked coastal ecosystems, nitrogen is the limiting element for primary production (Cunha & Wasserman, 2003; Rahav et al., 2018) and is rapidly assimilated by primary producers, exhibiting non-conservative behavior. In such nitrogen-limited environments, phosphorus behaves more conservatively and can serve as an effective tracer for sewage inputs (Borges et al., 2009). Conversely, in phosphate-limited environments, ammonium may be preserved and serve as a key indicator, alongside BOD. Regardless of the limiting factors, coastal cycling typically involves high sediment accumulation of both nutrients and elevated phytoplankton production (Carstensen et al., 2011; Church et al., 2006). While nutrient inputs from urban rivers vary seasonally with wastewater flow (Zhang et al., 2021), their concentrations in the sediments are less dynamic, reaching elevated, stable levels (Qin et al., 2019).

Biotic processes further modulate this cycle. Filtration in mussels can influence nutrient dynamics by transforming and translocating nutrients at ecologically relevant rates and contributing to eutrophication by mobilizing otherwise unavailable nutrients (Christensen et al., 2003; Williamson et al., 2018). Beneath mussel farms, sediment nutrient enrichment can be two to three times higher than in surrounding areas (Dahlback & Gunnarsson, 1981; Grant et al., 1995). However, the effects of intensive longline mussel aquaculture on benthic ecology, microbial mineralization, and nutrient fluxes remain poorly understood.

Given that eutrophication is a paramount water quality issue in coastal ecosystems, resulting from agricultural, industrial, and domestic activities, understanding these complex feedback loops is essential (Silva et al., 2019). Jurujuba Cove has been subject to particularly intense domestic wastewater inputs, hosting an area of intensive mussel farming, both vectors of degradation. This research aimed to evaluate physical and chemical factors governing porewater nutrient accumulation/release and sediment organic matter dynamics in an urban coastal system (Jurujuba Cove). The scientific question of the research is whether the sediment’s physicochemical conditions—shaped by currents, waves, and urban inputs or intensive mussel farming—determine organic matter and nutrient concentrations in the sediment and pore water.

## Materials and methods

### Study area

**Fig. 1:**
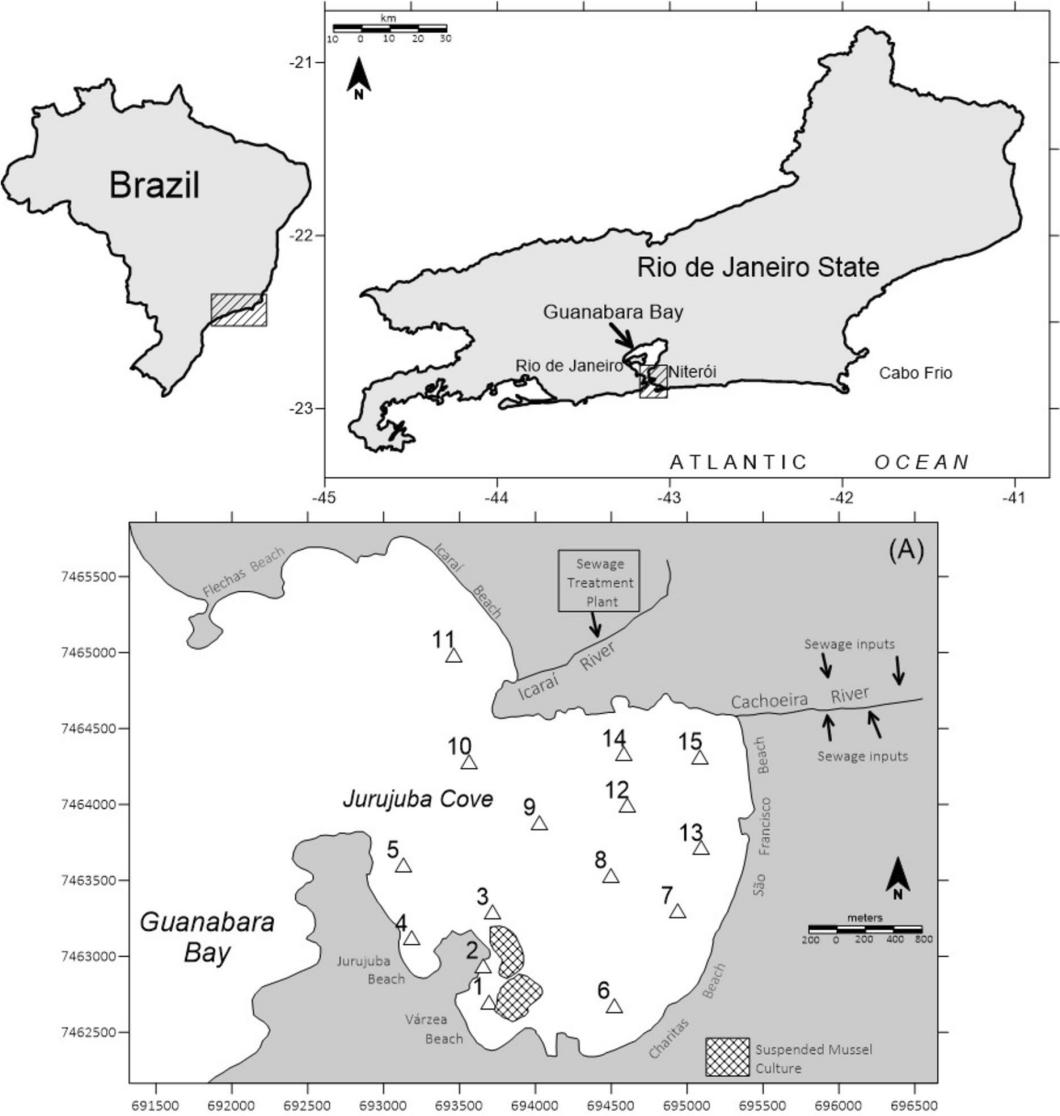
Location of Jurujuba Cove and sampling stations. Hatched figures indicate mussel farms. Coordinates in UTM (zone 23 K)

Guanabara Bay is a tropical environment located in the State of Rio de Janeiro, Brazil (Fig. 1), and is one of the largest bays in the country. The system is approximately 380 km^2^, with a volume of 1.87 × 10^9^m^3^ (Barrocas et al., 1995). The watershed comprises the municipalities of Rio de Janeiro, Mesquita, Nilópolis, Belford Roxo, Queimados, Duque de Caxias, Magé, Cachoeiras de Macaú, Guapimirim, Tanguá, Itaboraí, São Gonçalo, and Niterói, with more than 12 million inhabitants (IBGE, 2010). Jurujuba Cove, with approximately 7 km^2^, is located in the inner part of Guanabara Bay and is surrounded by the municipality of Niterói (Fig. 1). The currents are influenced by tides in the channel of the Bay, but velocities are extremely low inside the cove (not more than 20 cm s^−1^), which probably do not promote resuspension or remobilization in the sediment (Toste et al., 2024).

The Jurujuba Cove presents depths ranging from 5 to 7 m at the entrance of the cove and 3 to 4 m in the central part (Baptista Neto & da Silva, 1996). The Cachoreira and Icaraí rivers are the most important contributors to Jurujuba Cove. Despite easy water exchange with the bay entrance, the cove is severely affected by marinas and domestic sewage discharges. The site hosts the largest mariculture farms in the region, in an area of 100,000 m^2^, producing 216 tons of mussels per year (do Nascimento et al., 2018; Sabadini-Santos et al., 2014). After the Brazilian census 2010 (IBGE, 2010), the total population in the drainage basin of Jurujuba Cove is 156.003, who dispose wastewater that is partially treated by a primary sewage facility, the Icaraí Sewage Treatment Plant (Fig. 1). The treated wastewater is disposed of through an outfall in the middle of Guanabara Bay. During rain events, the mixed raw sewage/rainwater flows directly into the cove.

According to Fistarol et al. (2015), the inner waters of Jurujuba Cove are characterized by high nutrient loads, low concentrations of dissolved oxygen, and variable salinity, due to seasonal inputs of rainwater and sewage discharge. The outer parts of the system are generally characterized by higher salinities, lower nutrient levels, and elevated dissolved oxygen concentrations. These parameters form a distinct gradient of improving water quality toward the open Atlantic Ocean.

### Sampling

Fifteen surface sediment samples were collected with a Van Veen Grab Sampler during the winter of 2017 (3rd August 2017), with locations marked on a GPS, as described in Fig. 1. The sampler is a 1000 cm^2^ (25 × 40 cm) with a weight of around 8 kg, collecting layers between 5 and 8 cm. Although we recognize that there may be mixing and oxidation during the process, sediment sampling was carried out with care, reducing the effects of disturbance. In a recent laboratory assay, Vicente et al. (2022) had shown that an intensely reduced sediment oxidizes slowly when shaken in an open flask. The collected samples were placed in a tray where an aliquot was immediately placed in a pre-cleaned centrifuge flask (Falcon) and capped to avoid any modification of the redox condition. The samples were immediately refrigerated in an ice box and taken to the laboratory, where they were immediately prepared for centrifugation.

pH (with a combination electrode Hanna HI1210T) and redox potential were measured in the boat directly in the newly collected surface sediments with a Hanna® Multiparameter probe. pH was calibrated with pH 4, 7, and 10 buffer solutions prior to measurements, and temperature was simultaneously measured and automatically corrected. Eh electrode values were verified with a 240 mV reference solution.

### Sediment analysis

For grain-size analysis, the samples were prepared in three steps: first, they were oxidized with hydrogen peroxide to remove excess organic matter; secondly, they were washed with Milli-Q water; and thirdly, they were dried in an oven at 50 °C for 24 h. In the sequence, the samples were acidified with hydrochloric acid (HCl 1 M) to eliminate biological carbonates (mainly coccoliths). Particle-size distribution was determined with a laser granulometer (Malvern Instruments Mastersizer 2000) for grains < 1 mm.

Carbonate analysis was carried out with raw sediment aliquots between 0.5 and 2.2 g passed through a 1-mm sieve to exclude larger shells, then dried, weighed, and disposed of into 50-mL Falcon tubes. The sediments of the Falcon tubes were treated with a 10% HCl solution and shaken for at least 2 h until no further signs of reaction were visible. Afterward, each sample was washed three times with distilled water and centrifuged. After 24 h of drying, the samples were weighed again, and carbonate contents were determined as percentages.

The total phosphorus analysis was performed according to the method described by Aspila et al. (1976). To ensure analytical accuracy, the international reference material PACS-3 Marine sediment (National Research Council of Canada) was used, with a recovery rate of 75%. Although the recovery indicates underestimation of concentrations, the results were not corrected and were treated as not quite precise. The measurement of the extracted phosphate was performed in a Thermo Scientific™ GENESYS™ UV–Vis spectrophotometer at a wavelength of 880 nm. The detection limit (3 times standard deviation of 4 blanks) calculated for total phosphorus analysis was 34.9 µg g^−1^.

Organic matter was measured by loss on ignition of circa 3 g of sediment placed in porcelain crucibles. Samples were calcinated in a muffle furnace at 400 °C for 16 h. The measurement was gravimetric, and results were given as percentages.

Total organic carbon was measured by titrimetry after strong oxidation of the sample (with a sulfochromic solution). Titration was carried out with a ferrous sulfate solution (0.1 N), and ferroin was used as the indicator (Strickland & Parsons, 1972).

### Analyses of the interstitial water

Separation of the interstitial water was carried out in 50-mL capped Falcon tubes, which were filled with freshly collected sediment, then hermetically sealed to prevent redox alteration prior to analysis. The tubes were carefully weighed in groups of eight and centrifuged at 3000 rpm for 20 min. By capping the centrifugation flasks, we believe that it was possible to further reduce oxidation of the samples, considering that centrifugation promotes gravitational separation with very little shaking of the sample. Variable volumes were obtained, ranging from 5 to 15 mL, which were sufficient for the analyses.

For the analysis of dissolved phosphate, the method was based on the reaction of orthophosphate ions in an acidic medium with ammonium molybdate to form a phosphomolybdate complex. This complex was then reduced by ascorbic acid and catalyzed with potassium antimony tartrate to form a blue-colored complex (Hansen & Koroleff, 1999). A calibration curve with standard phosphate solution (10 mM) was constructed (2 µM, 4 µM, 6 µM, 10 µM, 20 µM, and 30 µM). A clear blue color developed, and the intensity of the light absorbance was measured with a KASUAKI UV/VIS spectrophotometer at a wavelength of 880 nm.

The analysis of ammonium was based on the method adapted from Hansen and Koroleff (1999), a spectrophotometric determination of the indophenol blue complex formed by the reaction of ammonia with phenol and hypochlorite in alkaline pH. A solution of NH_4_Cl (0.01 M) was prepared from the solid reagent, and six dilutions were prepared for the construction of a calibration curve (2 µM, 4 µM, 6 µM, 10 µM, 20 µM, and 30 µM). The intensity of the blue color was measured with a KASUAKI UV/VIS spectrophotometer with a wavelength of 630 nm.

Nitrate was determined by the resorcinol method developed by Zhang and Fischer (2006) for saline waters. For the calibration curve, a standard nitrate solution (0.01 M) was prepared with the same concentrations as described for the phosphate analyses. The intensity of the developed color was measured with a KASUAKI UV/VIS spectrophotometer with a wavelength of 505 nm.

Nitrite was analyzed after the method proposed by Shinn (1941) and adapted by Bendschneider and Robinson (1952), employing sulfanilamide and N-(1-naphthyl)ethylenediamine to develop a pink solution, whose intensity was measured in a spectrophotometer at a wavelength of 540 nm (KASUAKI UV/VIS spectrophotometer). For the calibration curve, a standard nitrite solution (0.01 M) was prepared, and the same concentration points as described in the phosphate and nitrate methodology were applied.

Four blank samples were carried out for each nutrient analysis, yielding detection limits (3 times standard deviation of blanks) of 3.1, 1.8, 80.0, and 7.6 µg L^−1^ for ammonium, nitrite, nitrate, and phosphate, respectively.

### Statistical analysis and distribution maps

In order to evaluate the dependency between behaviors of different variables, non-parametric Spearman correlation coefficients were calculated with the software Statistica® (Statsoft). No normality test was applied because the non-parametric correlation coefficient is a robust statistical test (Conover & Iman, 1981; Mardia et al., 1979). The statistical analyses were complemented with a cluster analysis in the Q-mode (samples) to allow the identification of spatial patterns in the Jurujuba Cove.

Contour distribution maps of concentrations and measured parameters were prepared with the interpolation method of kriging by blocks, 0th polynomial drift order, in the software SURFER® 13.3 (Golden Software).

## Results and discussions

### Physical and chemical parameters

**Fig. 2:**
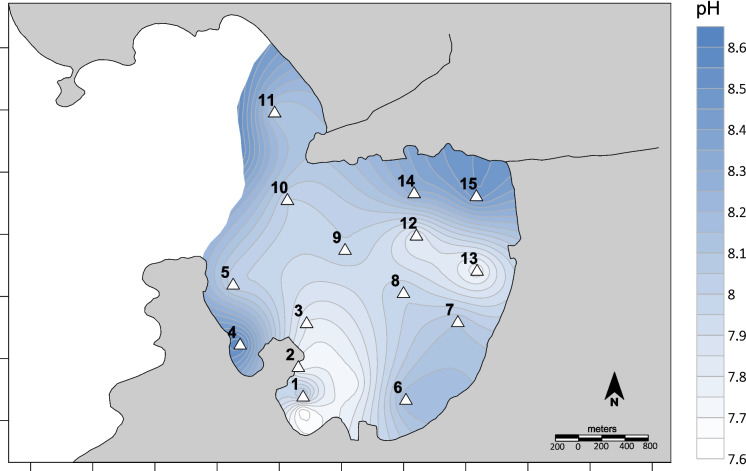
Spatial distribution of pH in Jurujuba Cove sediments’ porewater`

**Fig. 3:**
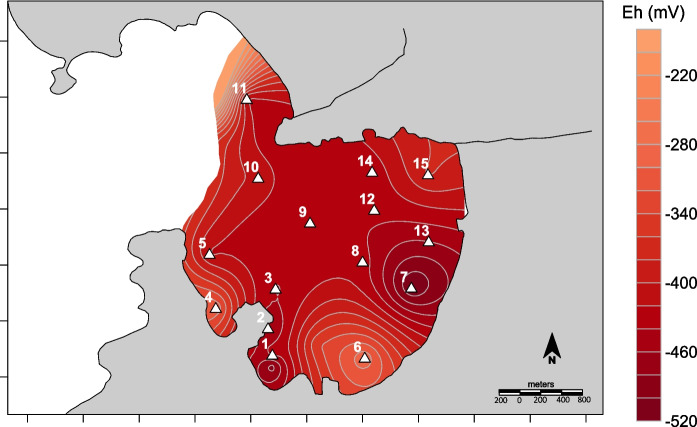
Spatial distribution of Eh in Jurujuba Cove superficial sediments

The sediment pH values ranged from 7.6 to 8.6, with the highest values recorded at stations 4 and 15 and the lowest at stations 2 and 13 (Fig. 2). In reducing sediments like those in Jurujuba Cove (Fig. 3), porewater pH is controlled by elevated dissolved organic carbon, lowering expected values from those of overlying seawater (~ 8.5) toward a neutral or slightly alkaline range (Curtin et al., 2022). However, in this study, porewater pH in many samples was unexpectedly more alkaline, with values closer to that of seawater. Recent studies have highlighted how porewater pH responds to variations in geochemical and microbiological processes, revealing that these systems are governed by complex interactions (Fouet et al., 2024). For instance, cable bacteria can significantly influence the electrogenic condition of sediments, driving poorly understood interactions and pH modifications (Burdorf et al., 2024). In the present research, pH serves as an accessory variable for inferring underlying geochemical processes within the sediments.

The spatial distribution of pH in Jurujuba Cove (Fig. 2) presented small variations. However, areas closer to the shore presented lower values (around 7.7) that could be associated with inputs of groundwater (Silva-Filho et al., 2009), together with the enrichment of organic matter. Inputs and preservation of organic matter are attributed to the presence of mussel farm structures that reduce the hydrodynamics, avoiding diffusion of oxygen into the sediments and acidifying pH (as shown in station 2).

Most of the reducing sediments (low Eh values) correlate with low pH values (*p* < 0.05; Supplementary Material 1). Although this is an unexpected behavior, other authors have observed the same correlation in similar conditions (Lopes et al., 2019; Luo et al., 2016). Negative Eh values were observed all over Jurujuba Cove, with the lowest values at Varzea Beach and Charitas Beach (in the Southwest of the Cove; Fig. 3).

The reduced hydrodynamics within the cove (Toste et al., 2024) likely inhibit the diffusion of dissolved oxygen produced by wind- and wave-induced mixing and algal photosynthesis (Olila & Reddy, 1997). When the “oxygenated” water diffuses into the pores of the sediments, the redox potential and pH increase (Araújo et al., 2009). Positive correlations between Eh and pH, and between Eh and sand grain size (*r* = 0.61 and 0.58, respectively) corroborate the above-mentioned behavior.

**Fig. 4:**
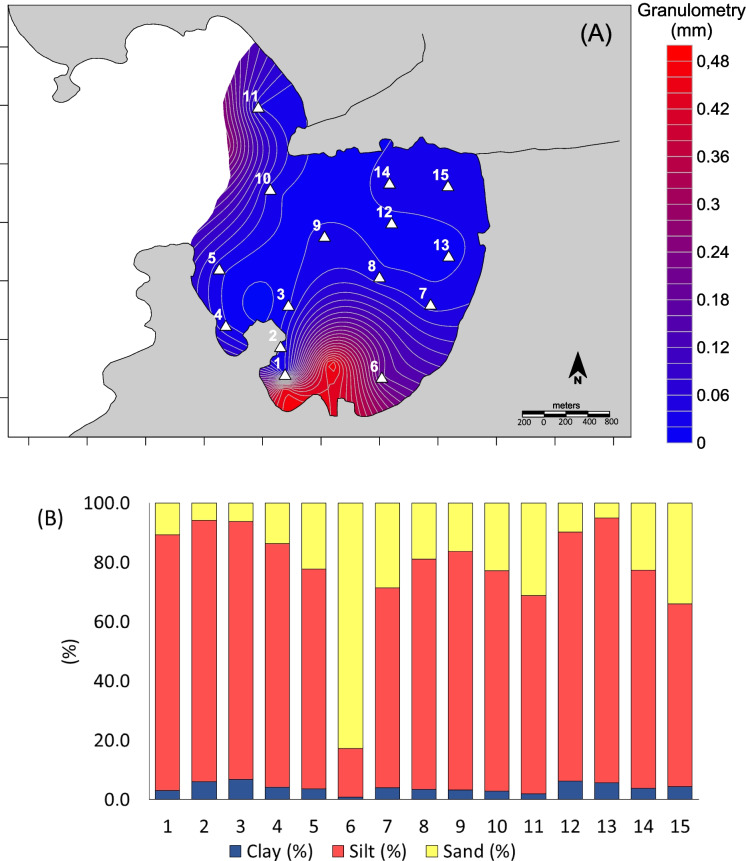
**A** Distribution map of the average grain size in Jurujuba Cove superficial sediments; **B** representation of percentages of grain size in each sample (%)

Jurujuba Cove tends to accumulate medium sands of approximately 0.4 mm, while areas closer to the Icaraí and Cachoeira river outlets are dominated by silts (Fig. 4A). These river inputs seem to contribute to the accumulation of fine sediments (silts of 0.02 mm) in the northern portion of the cove (Fig. 4A). The relationship between pH, Eh, and granulometry corroborated their close behavior, particularly noticeable in the neighborhood of station 6. As expected, stations 1, 2, and 3, within the area of the mussel farms, showed a finer granulometry (Fig. 4B) because the reduced hydrodynamics promote deposition of finer grains (Graca, 2009).

**Fig. 5:**
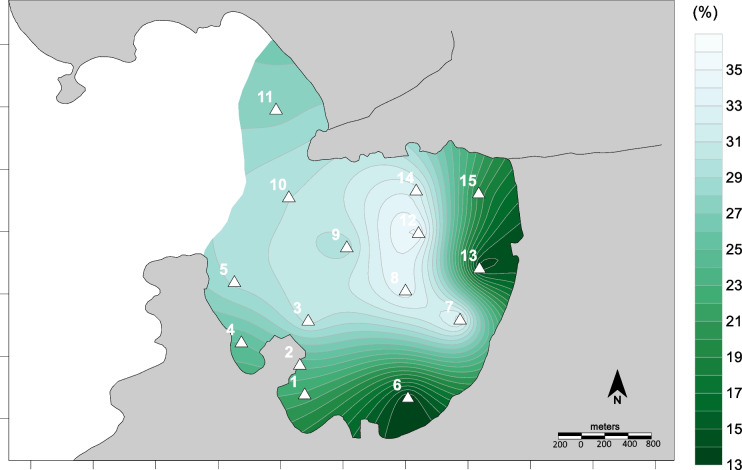
Spatial distribution of carbonates (CaCO_3_) in Jurujuba Cove superficial sediments

Carbonate percentages are consistently high across the entire system (Fig. 5), indicating both favorable chemical conditions for precipitation and significant external inputs. Lower values were observed near the mussel farms, associated with reduced pH, which reduces carbonate deposition. Furthermore, reduced hydrodynamics limits the input of smaller suspended particles and incorporation of carbonate-rich materials from the surrounding waters (Alonso-Pérez et al., 2010).

### Organic matter and total organic carbon

**Fig. 6:**
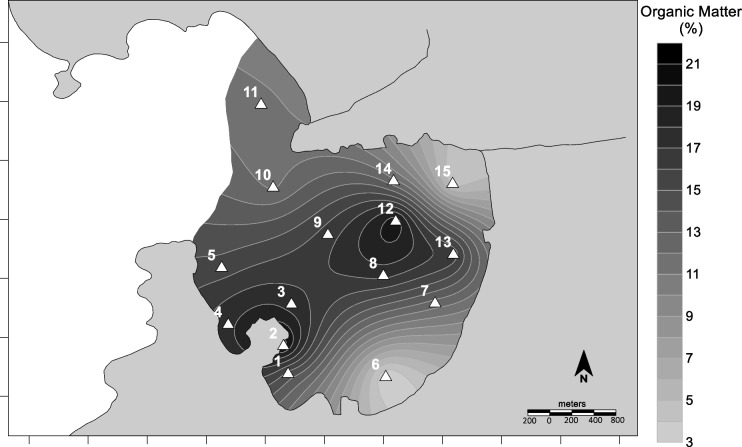
Spatial distribution of organic matter in Jurujuba Cove superficial sediments

The distribution map of percentages of organic matter (Fig. 6) indicated a similar behavior compared to the silt fraction (*r* = 0.82, *p* < 0.05; S.M. 1). The low-energy reducing environments are associated with the preservation of organic matter (Thompson & Eglinton, 1978) and the accumulation of fine-grained sediments. The preservation of organic matter is related to low dissolved oxygen and reducing conditions in porewaters, and inefficient anaerobic degradation cascades (Aguiar et al., 2013). Therefore, the organic matter concentrations in the sediments are linked to overlying water column dynamics that promote their preservation. Limited physical disturbance reduces resuspension and particulate organic matter release to the water column.

Even though the loss-on-ignition of sediments is broadly applied to evaluate organic matter (e.g., Burone et al. (2003)), the presence of hydrated minerals may overestimate concentrations, because hydrated minerals can bear water that is volatilized together with organic matter, during calcination (Sun et al., 2009). However, areas with small amounts of clay minerals should not present such a problem, and the procedure can be applied reliably.

**Fig. 7:**
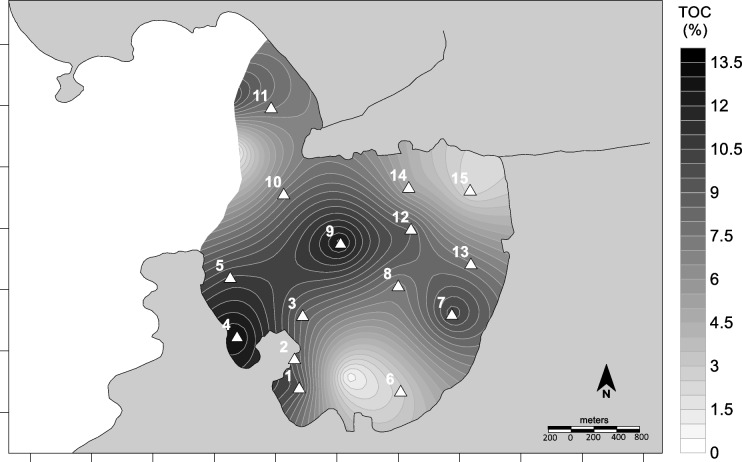
Total organic carbon distribution in Jurujuba Cove superficial sediments

As expected, the concentrations of total organic carbon (TOC) decreased with increasing grain sizes (Fig. 7), because larger grains are associated with higher energy, oxidizing conditions, and consequently low ability to preserve organic matter (Song et al., 2022). The preservation of TOC is not only promoted by the finer grain size but also by the anoxia of the sediments (Aguiar et al., 2013). On the other hand, smaller concentrations of TOC observed in the beaches of Charitas and São Francisco seem to be associated with breaking waves that, although small (30–40 cm), should promote aeration of the sediments and therefore oxidation of the organic matter.

### Nutrients

**Fig. 8:**
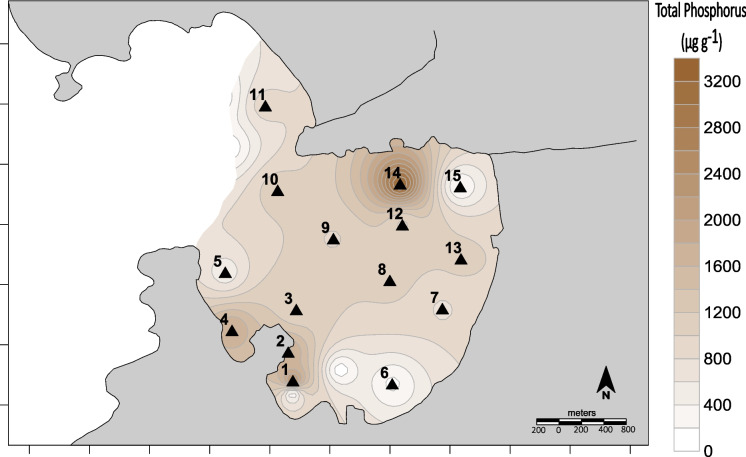
Spatial distribution of total phosphorus in Jurujuba Cove superficial sediments

The distribution of total phosphorus in surface sediments (Fig. 8) seems to be related to the inputs of domestic effluents. The highest values were found in the outlet of the Cachoeira River—circa 3 mg g^−1^. In Jurujuba Cove, total phosphorus can assume the role of a sewage indicator, due to its rather conservative behavior (Borges et al., 2009). Besides direct inputs from sewage, the accumulation of phosphorus may be associated with primary producers that consume dissolved phosphate (originating from sewage) and quickly recycle the organic phosphorus to the sediment (Carreira & Wagener, 1998).

**Fig. 9:**
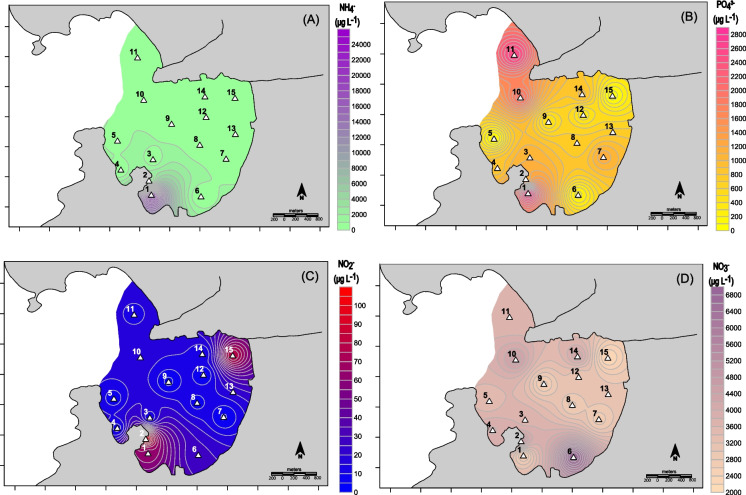
Spatial distribution maps of **A** ammonium, **B** phosphate, **C** nitrite, and **D** nitrate, in Jurujuba Cove porewater. Hatched figures indicate mussel farms

The highest ammonium concentration in the porewater was 23,000 µg L^−1^ (1278 µM), adjacent to the mussel farms area (Fig. 9A). The organic matter accumulation associated with anaerobic degradation provides a suitable condition for the enrichment of reduced species, such as ammonium (Zhu & Wang, 2011). The elevated ammonium concentration in this site can be explained by direct inputs from sewage and/or nitrate/nitrite reduction, but can also be associated with excreta from mussels. Depending on the station, different ammonium sources should have a stronger or smaller influence (Robertson & Groffman, 2007). The measurement of nitrogen isotopes (δ^15^N) should give a better understanding of these processes, allowing the identification of sources or processes that control their concentrations (Monteiro et al., 2012).

Mussels are efficient filter feeders, bioaccumulating nitrogen and phosphorus from the phytoplankton (Kang et al., 2018), which is excreted to the sediments. Near the mussel farms area, porewater presented the highest phosphate concentration, approximately 2500 µg L^−1^ (26 µM), which can be associated with mollusks’ excreta (Fig. 9B). However, Icaraí Beach also presented high levels, probably due to the domestic sewage treatment plant effluent that discharges into the Icaraí River (Fig. 1).

Nitrite, as an intermediate species of dissolved inorganic nitrogen, is typically present at lower concentrations in marine coastal sediments (Collos et al., 2003). As expected, nitrite indeed presented very low concentrations all over Jurujuba Cove (Fig. 9C). The Cachoeira River mouth and the mussel farms area presented higher concentrations, with approximately 80 μg L^−1^ (1.7 µM), probably due to enrichments from the excreta (Christensen et al., 2003).

Nitrate is the most stable species of dissolved inorganic nitrogen in oxidizing environments (De Caro et al., 2017; Matos et al., 2016; Schaefer & Hollibaugh, 2017). The highest nitrate concentrations were observed in the southwestern part of the cove, with approximately 6500 μg L^−1^ (135.4 µM) (Fig. 9D). The domestic effluents contribute ammonium to Jurujuba Cove, mainly from the Icaraí and Cachoeira rivers (Fig. 1), but oxidizing conditions and bacterial nitrification lead to conversion to nitrate (Aguiar et al., 2013).

Except for station 1, which presents very high concentrations of ammonium, all other stations showed a clear dominance of nitrate over ammonium and nitrite in the Jurujuba Cove. This behavior of nitrogen is not consistent with the reducing conditions of the Cove, as depicted in Fig. 3. Although sediments were largely reducing, nitrification may still occur in thin oxic microzones at the sediment–water interface or within bioturbated sediments, allowing conversion of ammonium to nitrate (Chen et al., 2021). Subsequent transport can enrich porewaters in nitrate even under overall low Eh conditions.

Increasing Eh in coarser sediments could be associated with the higher nitrate concentrations in the porewater. Therefore, when the ammonium produced in the mussel farms area or from sewage moves to higher Eh environments, it oxidizes to nitrite (unstable form) and then to nitrate (Liu et al., 2021).

### Cluster analysis

**Fig. 10:**
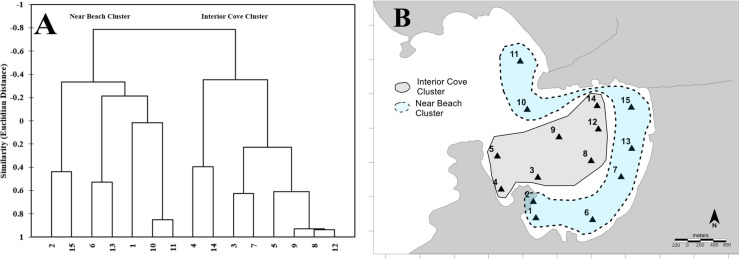
**A** The cluster analyses (Q mode) are divided into two main groups; **B** these two main groups are spatially represented, where the dashed line represents the near-beach station cluster, and the dotted line represents the interior–cove cluster

The cluster analyses showed the formation of two distinct groups, based on sediment characteristics (Fig. 10A). The near-shore samples represented an environment with higher oxidizing conditions associated with the incidence of breaking waves (Fig. 10B), illustrated by nitrate concentrations and related to sandy grain size. On the other hand, the middle of the cove cluster has reducing conditions favoring organic matter accumulation. The cluster analysis helped interpret sediment distribution, showing areas impacted by organic matter inputs, by grouping samples related to urban drainage and the suspended mussel culture.

## Conclusions

The mapping of sediment and porewater characteristics in Jurujuba Cove showed that the long-term input of organic matter and nutrients into this semi-confined environment leads to their accumulation. The slow degradation of organic matter controls concentrations in the sediments, while the reduced hydrodynamics determine redox potential and pH changes, favoring dissolved nitrogen accumulation.

Considering the extensive occupation in the drainage basin of the Icaraí and Cachoeira rivers, it is suggested that they constitute important sources of nutrients to the cove. Mussel farm sediments presented suitable conditions for the accumulation of nutrients and preservation of organic matter, due to a critical reduction of hydrodynamics and mollusk excreta contribution, leading to very high concentrations of ammonium in the porewater. The application of stable isotope analysis, particularly δ^15^N ratios, provides a means to trace and depict sources within the study area.

Breaking waves seem to be a local factor of oxidation of organic matter and the removal of nutrients from the sediments. The intensification of wave formation from boat traffic may expand oxidation of the sediments, affecting environmental quality and mussel farming. This is a relevant matter of conflict between mussel farmers and boat owners.

The diffusion of nutrients from porewater into the overlying water column may induce algal blooms and result in fish mortality. This diffusion largely depends on the system’s physicochemical conditions, and although diffusion models can provide predictions, in situ measurements remain crucial. Future studies involving bell jars (benthic chambers) or in vitro experiments would enhance our understanding of these processes.

Finally, sampling in this study was conducted during the winter season. This approach is justified by the fact that—unlike water and biota—sediment characteristics reflect long-term changes in settling materials. However, when interactions between overlying water and porewater are strong, seasonal variations may still occur, particularly during tropical rainy and dry periods. These potential variations could form the basis for further research, though such seasonal effects are likely relevant for porewater.

Because of the economic and touristic importance of this area, further detailed sediment and water quality studies are necessary, especially on the interactions of mussel cultivation with the water column and sediments. Although mussel farming, covering only 10 hectares, has a modest impact on the whole cove, it drains energy from the system and can be beneficial, especially if pseudo-feces are well managed.

## Supplementary Information

Below is the link to the electronic supplementary material.

Supplementary file1 (DOCX 30.9 KB)

## Data Availability

Most of the data were provided within the manuscript or supplementary information files. Further details can be provided under reasonable demand.
