# Supplementary material for: Factors determining nutrient distribution in sediments and porewater in a semi-confined coastal environment subject to anthropogenic pressure
Source: Environ Monit Assess. 2026 Feb 23;198(3):248. doi: 10.1007/s10661-026-15065-y (PMC12929330; doi:10.1007/s10661-026-15065-y)
Supplement: Supplementary file 1 — Supplementary file1 (DOCX 30.9 KB) [file 10661_2026_15065_MOESM1_ESM.docx]

**Factors determining nutrient distribution in sediments and porewater in a semi-confined coastal environment subject to anthropogenic pressure**

Gabriela Couger **de Pontes,** Susanne **Schmidt,** Murilo de Carvalho **Vicente,** Teresa Cristina **Guimarães,** Wilson Thadeu V. **Machado,** Julio Cesar **Wasserman**

**Supplementary Materials**

Supplementary Material 1: Spearman’s correlation matrix of variables. Highlighted correlations p<0.05

|  | Clay | Silt | Sand | CaCO3 | OM | Tot P | pH | Eh | NO3 | NO2 | NH4 |
| --- | --- | --- | --- | --- | --- | --- | --- | --- | --- | --- | --- |
| Silt | -0.1591 |  |  |  |  |  |  |  |  |  |  |
| Sand | 0.1679 | **-0.9902** |  |  |  |  |  |  |  |  |  |
| CaCO3 | -0.0840 | 0.0143 | -0.0572 |  |  |  |  |  |  |  |  |
| OM | -0.4179 | **0.8186** | **-0.8536** | 0.3146 |  |  |  |  |  |  |  |
| Tot P | 0.0643 | **0.5648** | **-0.5357** | 0.1644 | 0.4357 |  |  |  |  |  |  |
| pH | 0.2428 | **-0.6982** | **0.6975** | -0.1822 | **-0.5816** | -0.1069 |  |  |  |  |  |
| Eh | 0.1001 | **-0.5850** | **0.6095** | -0.3399 | -0.4969 | -0.2341 | **0.6565** |  |  |  |  |
| NO3 | -0.3714 | 0.1483 | -0.1500 | -0.2842 | 0.0464 | 0.1714 | -0.2337 | 0.0769 |  |  |  |
| NO2 | 0.0251 | -0.2206 | 0.2742 | -0.4323 | -0.3333 | 0.1703 | 0.2755 | 0.3776 | 0.1075 |  |  |
| NH4 | 0.1464 | -0.1001 | 0.0857 | -0.0357 | -0.1321 | 0.0536 | 0.2065 | -0.1251 | -0.4679 | 0.4086 |  |
| PO4 | 0.0321 | 0.1984 | -0.1250 | 0.1805 | -0.0107 | **0.5679** | -0.0399 | -0.2645 | 0.0429 | 0.2437 | 0.1357 |

Supplementary Material 2: Table showing raw data of this work. Including geographic coordinates (degrees and decimal degrees). Negative values indicate South (latitude) and West (Longitude).

| Coordinates | |  | Sediment | | | | | | Interstitial water | | | | | |
| --- | --- | --- | --- | --- | --- | --- | --- | --- | --- | --- | --- | --- | --- | --- |
| Latitude | Longitude | Stations | Clay (%) | Silt (%) | Sand (%) | CaCO_3_ (%) | OM (%) | Total P  (µg kg^-1^) | pH | Eh (mV) | NO_3_^-^  (µg L^-1^) | NO_2_^-^ (µg L-1) | NH_4_^+^ (µg L-1) | PO_4_^-3^ (µg L-1) |
| -22.9326 | -43.1112 | 1 | 8.0 | 86.2 | 10.7 | 22.2 | 15.1 | 2,353 | 8.1 | -433 | 2514 | 63.4 | 24,380 | 2,755 |
| -22.9304 | -43.1116 | 2 | 6.1 | 88.1 | 5.8 | 24.8 | 21.3 | 1,843 | 7.7 | -466 | 4680 | 102 | 240 | 1,095 |
| -22.9272 | -43.1110 | 3 | 6.8 | 87.1 | 6.1 | 31.3 | 17.9 | 1,061 | 7.8 | -441 | 3730 | 10.6 | 60 | 1,102 |
| -22.9288 | -43.1161 | 4 | 4.1 | 82.3 | 13.6 | 25.4 | 18.3 | 1,814 | 8.6 | -302 | 4020 | 7.92 | 46 | 842 |
| -22.9245 | -43.1167 | 5 | 3.7 | 74.2 | 22.1 | 29.9 | 15.8 | 486 | 8 | -422 | 3425 | 7.04 | 532 | 70 |
| -22.9327 | -43.1031 | 6 | 0.8 | 16.6 | 82.6 | 12.8 | 2.8 | 159 | 8.2 | -286 | 6810 | 25.5 | 16 | 162 |
| -22.9270 | -43.0991 | 7 | 4.0 | 67.4 | 28.5 | 34.6 | 13.0 | 778 | 8.1 | -522 | 2689 | 4.4 | 92 | 1,164 |
| -22.9249 | -43.1034 | 8 | 3.4 | 77.8 | 18.8 | 34.6 | 18.0 | 1,069 | 8 | -431 | 2756 | 7.92 | 5 | 706 |
| -22.9219 | -43.1080 | 9 | 3.3 | 80.5 | 16.2 | 30.0 | 17.0 | 987 | 8 | -439 | 2547 | 3.52 | 53 | 380 |
| -22.9183 | -43.1126 | 10 | 2.9 | 74.2 | 22.9 | 30.6 | 12.0 | 1,122 | 8 | -402 | 4509 | 16.7 | 40 | 1,964 |
| -22.9120 | -43.1137 | 11 | 1.9 | 66.8 | 31.3 | 27.9 | 11.7 | 899 | 8.1 | -420 | 3630 | 14.1 | 85 | 2,587 |
| -22.9208 | -43.1024 | 12 | 6.3 | 84.0 | 9.7 | 36.2 | 20.3 | 968 | 7.8 | -434 | 3023 | 4.4 | 56 | 322 |
| -22.9233 | -43.0976 | 13 | 5.7 | 89.2 | 5.0 | 13.1 | 17.3 | 1,043 | 7.7 | -459 | 5003 | 3.52 | <0.1 | 597 |
| -22.9177 | -43.1027 | 14 | 3.8 | 73.7 | 22.5 | 33.2 | 12.3 | 3,187 | 8.3 | -433 | 4159 | 10.6 | 150 | 928 |
| -22.9178 | -43.0978 | 15 | 4.3 | 61.7 | 34.0 | 19.8 | 4.0 | 218 | 8.5 | -378 | 2176 | 80.8 | 250 | 46 |
|  |  | Mean | 4.3 | 74.0 | 22.0 | 27.1 | 14.5 | 1,199 | 8.1 | -418 | 3711.4 | 24.2 | 1,858 | 981 |
|  |  | SD | 1.5 | 11.2 | 12.0 | 5.9 | 4.1 | 587 | 0.2 | 40 | 924 | 23.3 | 3,218 | 637 |

Supplementary Material 3: Rainfall record obtained from Barra/Barrinha meteorological station (<http://www.sistema-alerta-rio.com.br/dados-meteorologicos/download/dados-pluviometricos/>)
